# *AA*_*h*_ BN crystal, basic structure of boron nitride nanotubes

**DOI:** 10.1107/S2052252521009118

**Published:** 2021-10-16

**Authors:** Jae-Kap Lee, Jin-Gyu Kim, Seunggun Yu, Sang-Gil Lee, Yesong Kim, Dong Ju Moon

**Affiliations:** a Opto-Electronic Materials and Devices Research Center, Korea Institute of Science and Technology (KIST), Seoul, 02792, Republic of Korea; b Center for Scientific Instrumentation, Korea Basic Science Institute, Daejeon, 34133, Republic of Korea; c Insulation Materials Research Center, Korea Electrotechnology Research Institute, Changwon, 51543, Republic of Korea; d Center for Research Equipment, Korea Basic Science Institute, Daejeon, 34133, Republic of Korea; e Clean Energy Research Center (KIST), Seoul, 02792, Republic of Korea

**Keywords:** *AA*_*h*_ BN crystals, boron nitride nanotubes, morphology, transmission electron microscopy, electron diffraction, helical structures

## Abstract

*AA*_*h*_ boron nitride (BN) crystal, assigned to an orthorhombic space group (No. 31, *Pm*2_1_), is reported here. This new *AA*_*h*_ BN crystal exhibits a ‘linear’ morphology for high-resolution transmission electron microscopy (HRTEM) and a (non-hexagonal) ‘diagonal’ electron-diffraction pattern, which have been experimentally demonstrated in this article. It is also demonstrated that this new crystal is the basic structure of multi-walled BN nanotubes (BNNTs) existing in the form of a helix. The helical *AA*_*h*_ BNNTs exist in a metastable phase owing to 〈200〉 texture growth of the orthorhombic crystal, where the energy is ∼15 meV higher than that of stable *AB* or *AA′* BN. It is shown that the typical scanning electron microscope ‘fluffy cotton-like’ morphology of BNNTs is due to secondary growth of diverse BN sheets (including mono-layers) on incoherently scrolled wall strands of BNNTs, providing further evidence for the helical structure with HRTEM evidence for a left-handed helix.

## Abstract 2

For the first time, *AA*_*h*_ boron nitride (BN) crystal, assigned to an orthorhombic space group (No. 31, *Pm*2_1_), is reported. The structure of BN nanotubes is reinterpreted as a helix, resulting from texture and helical growth of the *AA*_*h*_ BN crystal used.

## 1. Introduction   

Synthetic boron nitride (BN) exhibits unique properties comparable in nature with those of graphite because of the similarity in crystal structure between these materials. Both materials feature a 2D van der Waals (*sp* ^2^) structure forming different crystalline structures, typically *AA* (or *AA′*) and *AB* (*AB′* or *A′B*) (Constantinescu *et al.*, 2013 ▸; Lee *et al.*, 2021 ▸), according to the stacking sequence of the planes. This structural similarity extends to multi-walled carbon nanotubes (MWNTs) and multi-walled BN nanotubes (BNNTs) (Iijima, 1991 ▸; Golberg *et al.*, 2010 ▸; Chopra *et al.*, 1995 ▸). It is generally understood that MWNTs are concentric tubes of graphene nanosheets with different diameters (Dresselhaus *et al.*, 1995 ▸). The tube model for MWNTs has also been accepted as the structure of BNNTs since their first report in 1995 (Chopra *et al.*, 1995 ▸) because the high-resolution transmission electron microscopy (HRTEM)-determined morphologies and X-ray diffraction (XRD) and electron diffraction (ED) patterns of the latter are very similar to those of MWNTs.

In a multi-walled tube structure, the stacking between concentric tube walls must be disordered. Thus, researchers have tried to explain the crystalline XRD and ED features of MWNTs and BNNTs in terms of chirality (Dresselhaus *et al.*, 1995 ▸; Ruland *et al.*, 2003 ▸; Meyer *et al.*, 2003 ▸) or periodic defects (Zhang *et al.*, 1993 ▸; Celik-Aktas *et al.*, 2005 ▸). However, Lee *et al.* (2013 ▸) showed that MWNTs are crystalline in helix structures resulting from (helical) textured growth of *AA′* graphite nanoribbons (see Fig. S1 of the supporting information), following the discovery of *AA′* graphite (Lee *et al.*, 2008 ▸). Lee *et al.* (2014 ▸) also reinterpreted the structure of a single-walled carbon nanotube (SWNT) as a graphene helix resulting from helical growth of a graphene nanoribbon and subsequent lateral growth of the helix (Park *et al.*, 2019 ▸).

BNNTs have been synthesized by plasma arc discharge, chemical vapour deposition (CVD) and laser-ablation methods, and are currently commercially available. The nanostructures reveal typical morphological features and XRD and ED patterns. The common and unique feature of BNNTs is a ‘fluffy cotton-like’ morphology (Kim *et al.*, 2014 ▸) observed in scanning electron microscopy (SEM) images (Smith *et al.*, 2009 ▸; Wang *et al.*, 2016 ▸; Kim *et al.*, 2019 ▸). Wang *et al.* (2016 ▸) attributed the SEM morphology to amorphous contaminants coated on the tube surfaces. Celik-Aktas *et al.* (2005 ▸) reported single-crystalline (revealed by the spot ED pattern) BNNTs, suggesting a double-helix model with a zigzag structure, as determined by their unique TEM morphology, which showed regular dark spots (revealing a helical trace) on BNNTs.

On the other hand, the existence of *AA′* stacked graphite as helical (MWNT) or ribbon structures (Lee *et al.*, 2013 ▸, 2016 ▸) suggests the possibility of the presence of another BN crystal, equivalent to *AA′* graphite. In this article, we reveal *AA_h_* stacked BN crystal and demonstrate that BNNTs are helical forms of this BN crystal with HRTEM and XRD measurements for commercial BNNTs.

## 2. Experimental   

We analysed commercial BNNTs (BNNT P1 Beta, LLC) synthesized by laser ablation developed by NASA. The samples were analysed by XRD (X’pert Pro, PANalytical) using a Cu *K_α_* source where the step size was 0.02°. Overall features of BNNTs were observed by SEM (field-emission SEM, Inspect F50, FEI) with an acceleration voltage of 10 kV, and their HRTEM imaging was performed by Cs-corrected TEM (Libra 200 HT Mc, Carl Zeiss) operating at 200 kV. The samples for SEM observation were prepared by placing the BNNT powders onto a carbon tape and sputtered with platinum that was nanometres thick. For TEM observations, the samples were dispersed in ethyl alcohol with sonication for 1 min, and the solution was then dropped onto a copper grid coated with lacey carbon films and air dried at room temperature. Selected-area electron diffraction (SAED) patterns were acquired from independent BNNTs. *DigitalMicrograph* (Gatan Inc.) software was used to analyse the HRTEM images and their fast Fourier transform (FFT) patterns. The interlayer binding energy for four different stacking structures of BN [Fig. S2(*a*)] was calculated by first-principles methods implemented in the *Quantum ESPRESSO* simulation package (Giannozzi *et al.*, 2009 ▸). Ultra-soft pseudopotentials were used to represent the interaction between ionic cores and valence electrons (Vanderbilt, 1990 ▸). A generalized gradient approximation was used for the exchange correlation energy of electrons (Perdew *et al.*, 1996 ▸). A plane-wave basis with an energy cut-off of 40 Ry was used with a suitable mesh of the grid (Monkhorst & Pack, 1976 ▸; Methfessel & Paxton, 1989 ▸). XRD patterns of all the structures were generated by the *FullProf* suite for comparison with the experimental data (Rodríguez-Carvajal, 2001 ▸).

## 3. Results and discussion   

### 3.1. *AA_h_* BN crystal   

**Figure 1:**
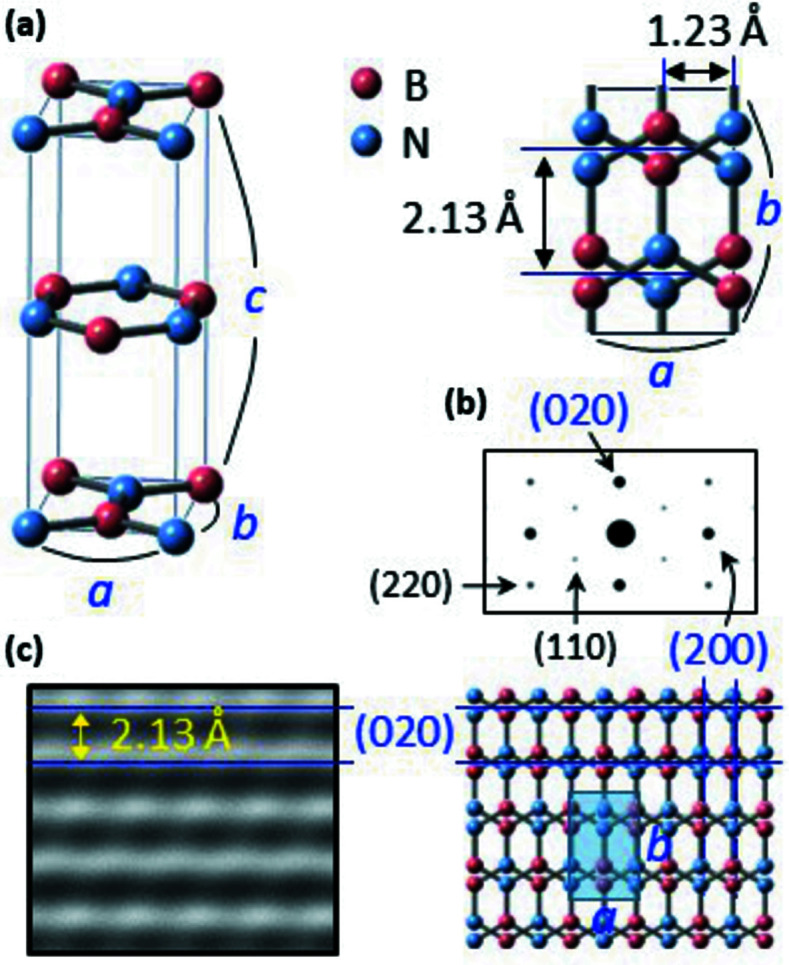
The crystal structure of *AA_h_* BN. (*a*) A crystal unit of *AA_h_* BN assigned as orthorhombic (No. 31, *Pm*2_1_). (*b*) A simulated ED pattern of *AA_h_* BN. Strong (020) and (200) spots result in a diagonal pattern, although relatively weak (110) and (220) spots appear. (*c*) A simulated HRTEM image of *AA_h_* BN. Linear lattices, corresponding to (020), are evident.

Fig. 1 ▸(*a*) shows the crystal unit of *AA_h_* BN, assigned to an orthorhombic structure (No. 31, *Pm*2_1_) where *a*, *b* and *c* are 2.46 Å, 4.26 Å and 6.88 Å, respectively. Our *AA_h_* BN can be defined as a horizontal shift of every other BN plane by a half hexagon (∼1.23 Å) relative to the *AA* zigzag structure or as a vertical shift by a half interatomic distance (∼0.71 Å) relative to the *AB* zigzag structure (Fig. S2). As shown in Figs. 1 ▸(*b*) and 1 ▸(*c*), the crystal exhibits a diagonal ED pattern and a linear HRTEM morphology.

**Figure 2:**
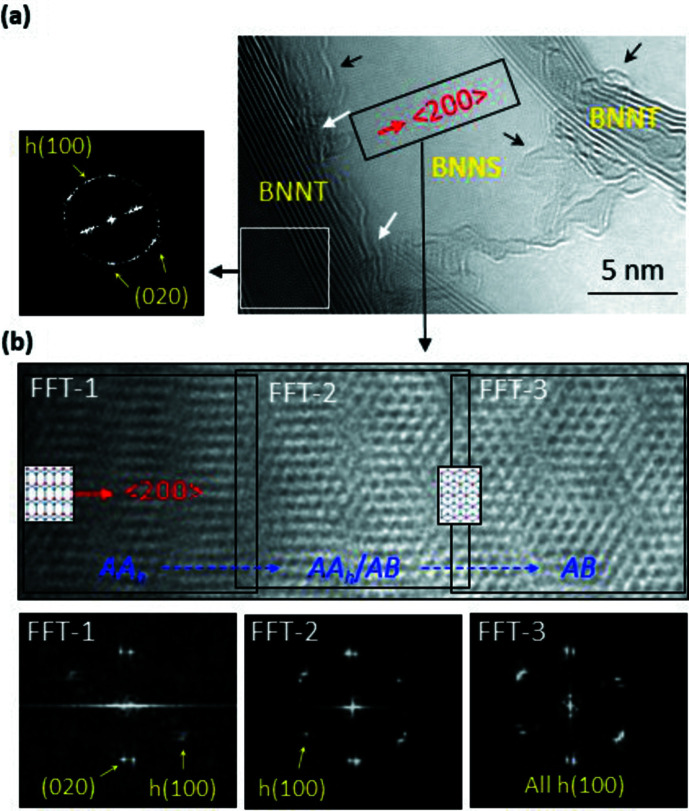
HRTEM data of a BNNT sample. (*a*) An HRTEM image of the BNNT sample, revealing the BNNT–BNNS structure where the BNNS grew from the BNNT on the left. (*b*) An atomic resolution image of the rectangle in (*a*). Split spots in the FFT patterns indicate that the BNNS consists of two BNNSs twisted by a few degrees. *h*(100) spots are of the hexagonal structure due to local sliding of the BN sheets destroying the orthorhombic structure.

The linear lattices, corresponding to (020) of orthorhombic *AA_h_* BN, are evident in the atomic resolution image of a BN nanosheet (BNNS) [Fig. 2 ▸(*b*)]. The analysis is proven by the unique (non-hexagonal) FFT pattern for *AA_h_* (FFT-1) where the strong (020) spots are evident with *h*(100) (which is due to local sliding of BN planes collapsing the orthorhombic crystal). Hexagonal HRTEM morphology for *AB* BN, appearing as Morrie pattern (disorderly overlapped *AB*/*AB* BN), is also evident in FFT-3. The data demonstrate the presence of *AA_h_* BN in nature which clearly differs with *AB* (or *AA*) BN in hexagonal symmetries. We also point out that the BNNS is interconnected to the left BNNT [see the white arrows in Fig. 2 ▸(*a*)].

### 3.2. Structure of BNNTs: *AA_h_* BN crystal based helix   

**Figure 3:**
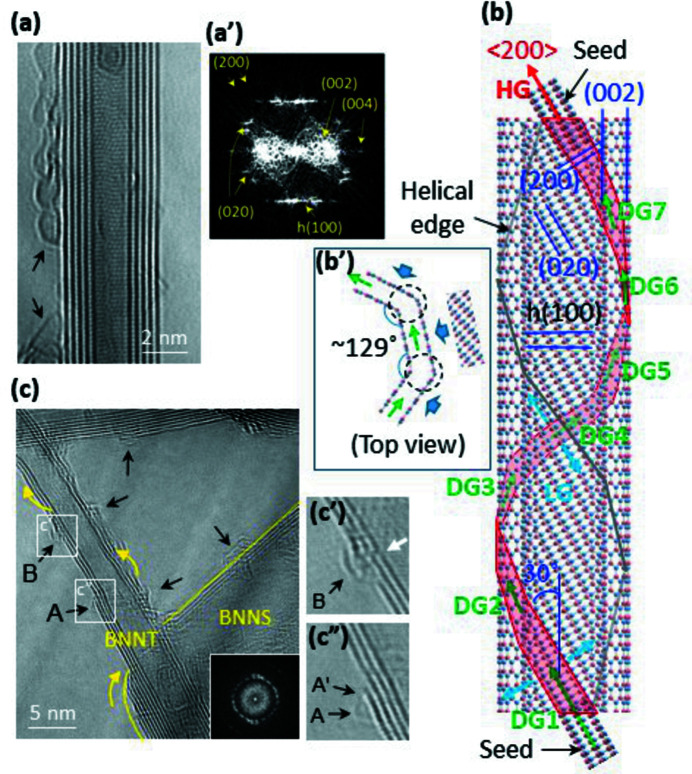
HRTEM images and the helical-growth model of BNNTs. (*a*) An HRTEM image of a BNNT. (*a′*) The FFT pattern obtained from the HRTEM image in (*a*). (*b*) The helical-growth model for a BNNT (*AA_h_* BN helix with a zigzag structure) based on those of MWNTs (Lee *et al.*, 2013 ▸) and SWNTs (Lee *et al.*, 2014 ▸). An *AA_h_* BN nanoribbon seed (bottom) initiates and leads to helical growth comprising seven diagonal growths, which is assumed to adopt the heptagon cross-sectional morphology of an MWNT (Fig. S1). HG, DG and LG in (*b*) indicate helical growth, diagonal growth and lateral growth, respectively. (*b′*) A schematic explaining the facet growth of BN layers to maintain crystallinity. (*c*) An HRTEM image of a BNNT sample. The yellow arrows indicate left-handed scrolling. (*c′*), (*c′′*) Magnified images of the squares in (*c*). A′ in (*c′′*) indicates a disconnected layer. The white arrow in (*c′*) indicates helical edges where all of the wall layers are disconnected. The black arrows in (*a*) and (*c*) indicate the traces of mono-layer BN.

HRTEM images of the BNNT samples are shown in Fig. 3 ▸. Well developed BNNT with a diameter of ∼5 nm [Fig. 3 ▸(*a′*)] reveals an FFT pattern which is identical to the expected ED pattern for an *AA_h_* zigzag BN existing in helix where (020) and (200) spots appear with the (002), (004) and *h*(100) spots (Fig. S3). The other two BNNTs also reveal SAED patterns consistent with the zigzag helix structure (Fig. S4). The HRTEM data indicate that the BNNTs adopt *AA_h_* zigzag BN in the form of a helix. The unique geometry of BNNT and BNNS structures shown in Fig. 3 ▸(*c*), in which the BNNS is placed beneath the BNNT and spreads out from left to right, provides evidence that the helix has formed via a left-handed scroll.

In the helix model for BNNTs, lateral growth following helical growth of an *AA_h_* BN nanoribbon seed creates a tube-like structure, as depicted in Fig. 3 ▸(*b*). Parallel bending of the BN layers [Fig. 3 ▸(*b′*)] allows the polygonal helix to maintain its crystallinity. The crystallinity, however, may not be perfect throughout the helix because spiral growth of layered materials naturally accompanies a slight sliding of the layers, thus partially collapsing the order in stacking. These results explain the observation of *h*(100) spots in the FFT [Fig. 3 ▸(*a′*)] and ED patterns of BNNTs (Fig. S4). We attribute the appearance of twin spots [Fig. 3 ▸(*a′*)] to a horizontal tilt (measured to be ∼3°) of the crystalline tube-like sample on the TEM grid (Zhang & Amelinckx, 1994 ▸; Lee *et al.*, 2013 ▸) rather than to chirality.

Many well defined SAED patterns of BNNTs, which are identical to those analysed in this work, have been previously reported from diverse samples prepared by CVD (Celik-Aktas *et al.*, 2005 ▸; Wang *et al.*, 2016 ▸; Golberg *et al.*, 2000 ▸), laser ablation (Arenal *et al.*, 2006 ▸) and arc deposition (Kim *et al.*, 2014 ▸; Demczyk *et al.*, 2001 ▸). The unassigned SAED spots reported by Celik-Aktas *et al.* (2005 ▸) can be explained as those for *AA_h_* BN. These indicate that conventional BNNTs have a helical structure. We expect that a right-handed helical scroll may be possible according to the initial (left- or right-handed) curvature of the nanoribbon nuclei, although we have shown evidence for left-handed helical growth in this work [Fig. 3 ▸(*c*)].

### 3.3. Morphological features of BNNTs   

**Figure 4:**
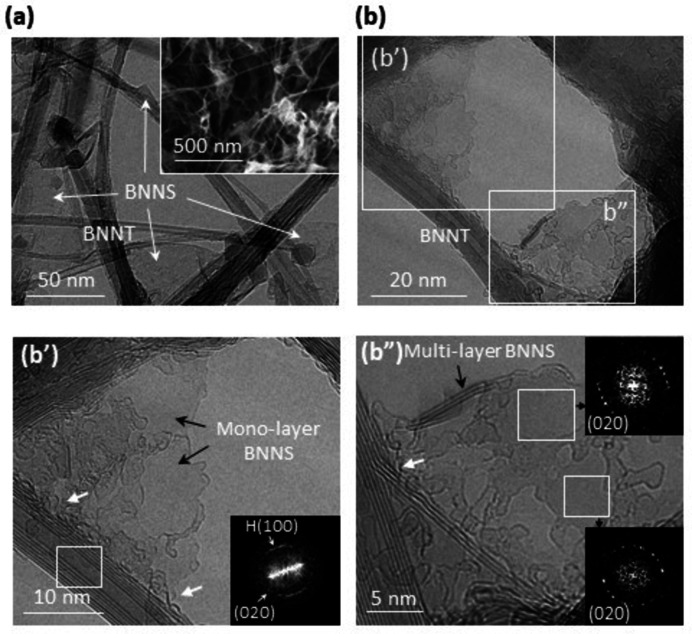
HRTEM images of BNNTs. (*a*) A low-magnification TEM image of the BNNT sample. The inset shows an SEM image revealing the typical fluffy cotton-like morphology of BNNTs. (*b*) An HRTEM image revealing the typical fluffy cotton-like morphology of BNNTs with coexisting mono-layer and multi-layer BNNSs. (*b′*), (*b′′*) Magnified images in the squares in (*b*). The inset in (*b′*) shows the FFT pattern obtained from the square on the left BNNT, revealing evidence for the zigzag *AA_h_* BN helix. The multi-layer BNNS (*b′′*) reveals the unique eye-like FFT patterns for *AA_h_* BN (insets).

Fig. 4 ▸(*a*) shows a low-magnification TEM image of laser-ablated BNNT samples. The fluffy cotton-like SEM morphology unique to BNNTs (Smith *et al.*, 2009 ▸; Wang *et al.*, 2016 ▸; Kim *et al.*, 2019 ▸) is evident in the SEM image of the sample [inset in Fig. 4 ▸(*a*)]. A fluffy cotton-like structure is imaged by HRTEM [Fig. 4 ▸(*b*)] where a BNNT coexists with mono-layer and multi-layer BNNSs. The multi-layer BNNS reveals eye-like FFT patterns [insets in Fig. 4 ▸(*b′′*)]. Such unique FFT patterns appear when *AA_h_* BNNSs overlap in a twisted manner, while twisted overlapping of *AB* BNNSs results in a ring pattern (Fig. S5), thus providing evidence for the presence of *AA_h_* BN. The unique HRTEM morphology, in which the multi-layer BNNSs spread out from the wall of the *AA_h_* BNNTs [Fig. 4 ▸(*b′′*)], indicates that the BNNSs grew further after branching from the wall [the white arrows in Figs. 4 ▸(*b′*) and 4(*b′′*)], maintaining the inherent *AA_h_* stacking.

The HRTEM data (Fig. 4 ▸) explain the BNNT–BNNS interconnect morphology, shown in Fig. 2 ▸(*a*), by showing that the BNNS has branched from the left BNNT and has grown to the right via the preferred 〈200〉 oriented growth. The analysis regarding the BNNT–BNNS structures [Figs. 2 ▸(*a*) and 4 ▸(*b*)] explains the unique morphology observed in Fig. 3 ▸(*c*), where the BNNT is lacking four layers of the wall and the BNNS contains the four layers. The morphology indicates that the four layers released from the wall grew further to the BNNS during laser ablation. The FFT pattern of the BNNS revealing an uneven ring pattern [inset in Fig. 3 ▸(*c*)] indicates that the sheet consists of disorderly overlapped *AA_h_* BN. The data indicate that the fluffy cotton-like SEM morphology of BNNTs is due to their coexistence with diverse BN sheets with a lateral dimension of approximately hundreds of nanometres.

The left-handed helical BNNT reveals several traces of mono-layer BN, as indicated by the arrows in Fig. 3 ▸(*c*). Practically, all the BNNTs we analysed in this work revealed evidence for mono-layer BN [Figs. 2 ▸(*a*), 3 ▸(*a*), 3 ▸(*c*), 4 ▸(*b′*) and S6]. Mono-layer BN is very rare, even in the CVD approach for the synthesis of 2D BNNSs on catalytic transition metal substrates (Sutter *et al.*, 2013 ▸; Khan *et al.*, 2017 ▸). We attribute the prevalence of mono-layer BN to the helical structure of the BNNTs. Here, there are two possible origins of the mono-layer BN on the surface of the helical BNNTs: the breakaway of the outermost layer [arrow A′ in Fig. 3 ▸(*c′′*)] on which the mono-layer BN grows further and mismatched helical edges (*i.e.* helical defects), which serve as a seed for mono-layer BN [arrow B in Fig. 3 ▸(*c*)] instead of the breakaway layer. The former may deploy a mono-layer BNNS similar to the multi-layer BNNS [Figs. 2 ▸(*a*), 3 ▸(*c*) and 4 ▸(*b′′*)], while the latter may generate localized mono-layer BN bound on the surface of BNNTs. This explains the appearance of the mono-layer BNNSs [Fig. 4 ▸(*b′*)] as well as the ubiquitous traces of mono-layer BN on BNNTs. Our analysis, based on HRTEM observations, clarifies that the typical fluffy cotton-like SEM morphology of BNNTs is due to BNNSs (including mono-layers) atomically interconnected to the helical BNNTs. This indicates that conventional BNNTs have a helical structure.

We expect that the fluffy cotton-like morphology is comparable with the bamboo morphology that is unique to MWNTs (Lee *et al.*, 2013 ▸; Jia *et al.*, 2017 ▸), Both are due to the release of the nanoribbons comprising the helix wall during spiral growth. Inward release of the *AA′* graphite ribbons produces the bamboo structure of MWNTs, while outward release (and further growth) of the *AA_h_* BN ribbons produces the typical fluffy cotton-like structure for BNNTs. We attribute the difference to the ∼2.5-fold higher bending stiffness of BN layers (acting as a resistance to the spiral growth) than of graphene layers (Qu *et al.*, 2019 ▸). Similarly, the dominant mono-layer BN is comparable with the case of MWNTs, on which traces of graphene are frequently observed (Jia *et al.*, 2017 ▸; Gerard Lavin *et al.*, 2002 ▸). We suggest that the graphene layers observed in contact with MWNTs are overgrown and indivisible from their helical edges, thus providing evidence for their helical structure. We infer that the regular dark spots on BNNTs reported by Celik-Aktas *et al.* (2005 ▸) may be due to BNNSs (which may be mono-layers) grown on their helical edge (*i.e.* the unique dark spots on BNNTs have a kind of fluffy cotton-like morphology analysed in Fig. 4 ▸). The perfectness of BNNTs varies depending on the synthesis methods and conditions used, and some coherently scrolled BNNTs can be seen as a concentric tube with a clean surface, explaining their diverse surface states (Celik-Aktas *et al.*, 2005 ▸; Kim *et al.*, 2014 ▸; Golberg *et al.*, 2000 ▸; Arenal *et al.*, 2006 ▸; Demczyk *et al.*, 2001 ▸; Bechelany *et al.*, 2008 ▸; Allard *et al.*, 2020 ▸).

### 3.4. XRD analysis of BNNTs sample   

**Figure 5:**
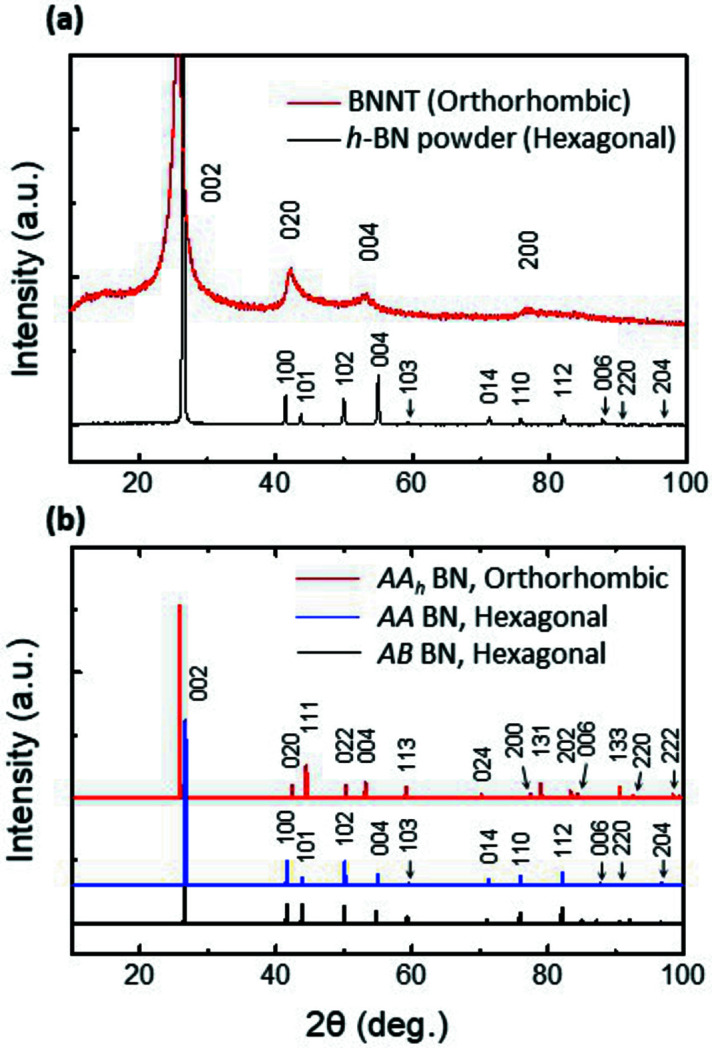
XRD analysis for BN structures. (*a*) XRD patterns of BNNTs and BN powders. The (020) and (200) peaks of the BNNTs (orthorhombic) are equivalent to (100) and (110) of the BN powders (hexagonal), respectively. (*b*) Simulated XRD patterns of typical BN crystals. *AA_h_* BN adopts an orthorhombic structure, while *AA* and *AB* BN (where *a*, *b* and *c* are 2.46 Å, 2.46 Å and 6.70 Å, respectively) adopt a hexagonal structure.

**Figure 6:**
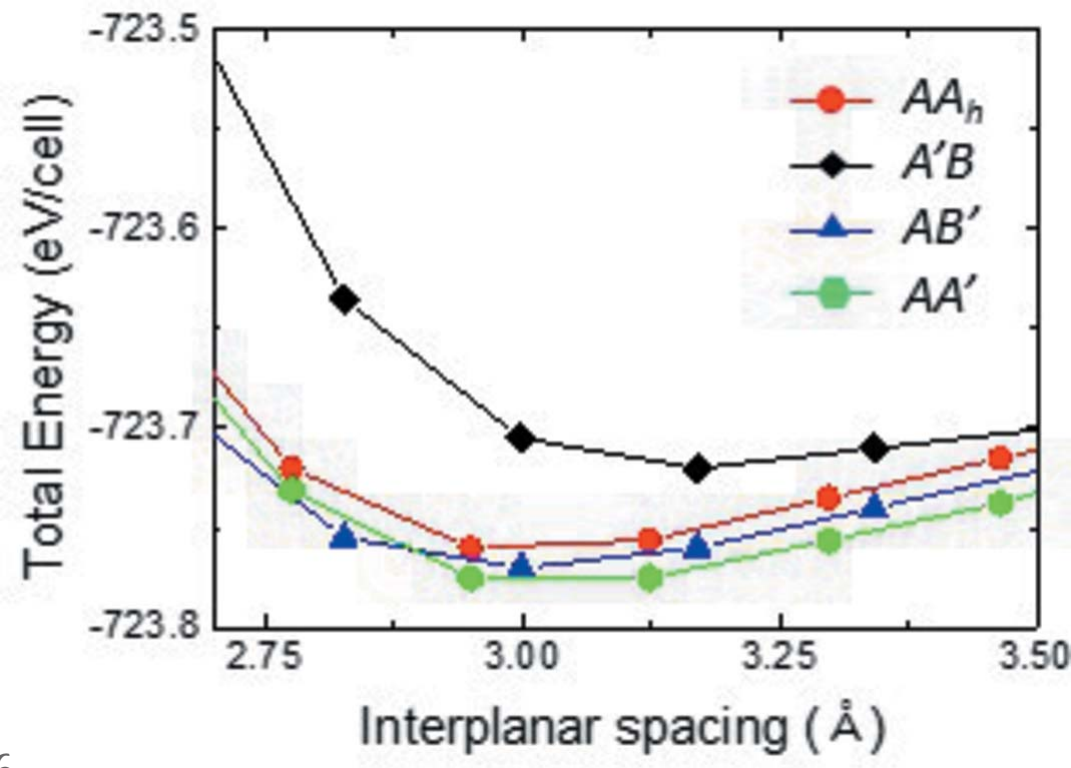
Calculated stacking energies with stacking structures for bi-layer *h*-BN. *AA′* stacking is the most stable configuration for BN.

The unique XRD pattern for BNNTs, exhibiting four peaks including (002) at 2θ ≃ 25.8° (3.45 Å), (020) at 2θ ≃ 42° (2.13 Å), (004) at 2θ ≃ 53° (1.72 Å), and (200) at 2θ ≃ 77° (1.23 Å), is evident in Fig. 5 ▸. The (helical) texture growth of *AA_h_* BN nanoribbon seeds [Fig. 3 ▸(*b*)] can explain the unique pattern, lacking several peaks of commercial BN powders [black line in Fig. 6 ▸(*a*)] which were determined to be *AB* (or *AA′*) BN (Warner *et al.*, 2010 ▸; Sutter *et al.*, 2013 ▸; Khan *et al.*, 2017 ▸). The interplanar spacing was measured to be 3.45 Å, which is larger than that (3.35 Å) of BN powders and is close to the value of 3.44 Å for *AA′* graphite. The data indicate that the BNNT sample has a textured *AA_h_* BN structure, similar to that of MWNTs. With texturing, the (*hk*0) (0*kl*), (*h*0*l*) and (*hkl*) peaks become weaker, while the (*h*00) and (0*k*0) peaks become stronger (Lee *et al.*, 2014 ▸, 2016 ▸), compared with the simulated pattern for *AA_h_* BN [Fig. 6 ▸(*b*)]. On the other hand, the broad XRD (002) peak for BNNTs (da Silva *et al.*, 2018 ▸; Harrison *et al.*, 2019 ▸) can be explained by the (helical) facetted growth of the BNNTs where any ordered stacking may not be retained at the corners of the helix [dotted circles in Fig. 3 ▸(*b′*)]. The XRD pattern of BNNTs (Fig. 5 ▸) is typical for BNNTs (da Silva *et al.*, 2018 ▸; Harrison *et al.*, 2019 ▸). These indicate that our helix model can also be explained by XRD analysis.

### 3.5. Energy calculation   

The interlayer binding energy for different stacking structures of BN is shown in Fig. 6 ▸. The energy of *AA_h_* BN is ∼15 meV higher than that of *AB* (*AB′*) or *AA′* BN (Constantinescu *et al.*, 2013 ▸; Topsakal *et al.*, 2009 ▸; Gilbert *et al.*, 2019 ▸) and ∼40 meV lower than that of unstable *A′B* BN, indicating that our unique *AA_h_* BN crystal can be defined as a metastable phase of BN. This is similar to the case of graphite, in which *AA′* stacking is a metastable phase with an energy ∼1.1 meV higher than that of *AB* BN (Lee *et al.*, 2016 ▸). The existence of metastable *AA_h_* BNNTs (kinetically formed by texture growth) may be due to their diagonal facets, which prohibit the change to stable *AB* or *AA′* BN. We attribute the formation of the metastable *AA_h_* BN phase (Fig. 6 ▸) to the 〈200〉 preferred growth orientation [Fig. 3 ▸(*b*)] due to the anisotropic stacking of the BN planes (Fig. 1 ▸). With texture growth, the metastable phase can be competitively compared with *AB* (or *AA′*) stacking (Lee *et al.*, 2016 ▸).

### 3.6. Observation of heterostrain   

Huder *et al.* (2018 ▸) reported heterostrain of layered (graphene) materials where each layer is strained independently. We attribute the panoramic variation of the BNNS, shown in Fig. 2 ▸(*b*), to heterostrain (originated from a nano-curvature of the BNNT) twisting or slipping of mono-layer or few-layer sheets. The coexistence of *AA_h_* BN and *AB* BN structure in the BNNS is due to gradual phase transformation of the BNNS from *AA_h_* (FFT-1) to *AB* (FFT-3) through the uneven hexagonal pattern in the transition stage (FFT-2) [Fig. 2 ▸(*b*)]. This analysis, based on the heterostrain, may explain the twisted overlap of the *AA_h_* BN sheets (evident by the unique eye FFT pattern) [Fig. 4 ▸(*b′′*)] atomically bonded to the BNNT. The HRTEM and FFT data, shown in Fig. 2 ▸, demonstrate the presence of *AA_h_* BN as well as the presence of heterostrain in layered materials.

## 4. Summary   

In summary, we have reported on *AA_h_* BN crystal assigned to an orthorhombic space group (No. 31, *Pm*2_1_). Our crystal appears as the helical structure of BNNTs, resulting from the spiral texture growth of the anisotropic stacking structure of the orthorhombic *AA_h_* BN crystal. The fluffy cotton-like morphology typical to BNNTs is due to incoherent scroll of the BNNSs. Heterostrain in layered materials is demonstrated, resulting in localized transformation of *AA_h_* BN to *AB* BN of which coexistence in a BN sheet is imaged by HRTEM. The ubiquitous traces of mono-layer BN on BNNTs become general evidence of our helical structure model. Our analysis shows that the structure of BNNTs should be interpreted based on crystalline growth, which occurs along the minimum energy path.

## Supplementary Material

Crystal structure: contains datablock(s) I. DOI: 10.1107/S2052252521009118/ct5015sup1.cif

Supporting information. DOI: 10.1107/S2052252521009118/ct5015sup2.pdf

Structure factors as generated with CrystalMaker. DOI: 10.1107/S2052252521009118/ct5015sup3.txt

CCDC reference: 2107341
