# Supplementary material for: AA h BN crystal, basic structure of boron nitride nanotubes
Source: IUCrJ. 2021 Oct 16;8(Pt 6):1018–23. doi: 10.1107/S2052252521009118 (PMC8562660; doi:10.1107/S2052252521009118)
Supplement: Supplementary file 2 [file m-08-01018-sup2.pdf]

IUCrJ

Volume 8 (2021)

Supporting information for article:

AAh BN crystal, basic structure of boron nitride nanotubes

Jae-Kap Lee, Jin-Gyu Kim, Seunggun Yu, Sang-Gil Lee, Yesong Kim and Dong Ju Moon

Supporting Information for

AA_h BN Crystal, Basic Structure of Boron Nitride Nanotubes

Jae-Kap Lee,[a]* Jin-Gyu Kim,[b] Seunggun Yu,[c] Sang-Gil Lee,[d] Yesong Kim,[a] and Dong-Ju Moon[e]

[a]Opto-Electronic Materials and Devices Research Center, Korea Institute of Science and Technology (KIST), Seoul 02792, Republic of Korea

[b]Center for Scientific Instrumentation, Korea Basic Science Institute, Daejeon 34133, Republic of Korea

[c]Insulation Materials Research Center, Korea Electrotechnology Research Institute, Changwon 51543, Republic of Korea

[d]Center for Research Equipment, Korea Basic Science Institute, Daejeon 34133, Republic of Korea

[e]Clean Energy Research Center, KIST, Seoul 02792, Republic of Korea

*Corresponding author: Jae-Kap Lee (jklee@kist.re.kr)

This PDF file includes:

- Figures S1 to S6.

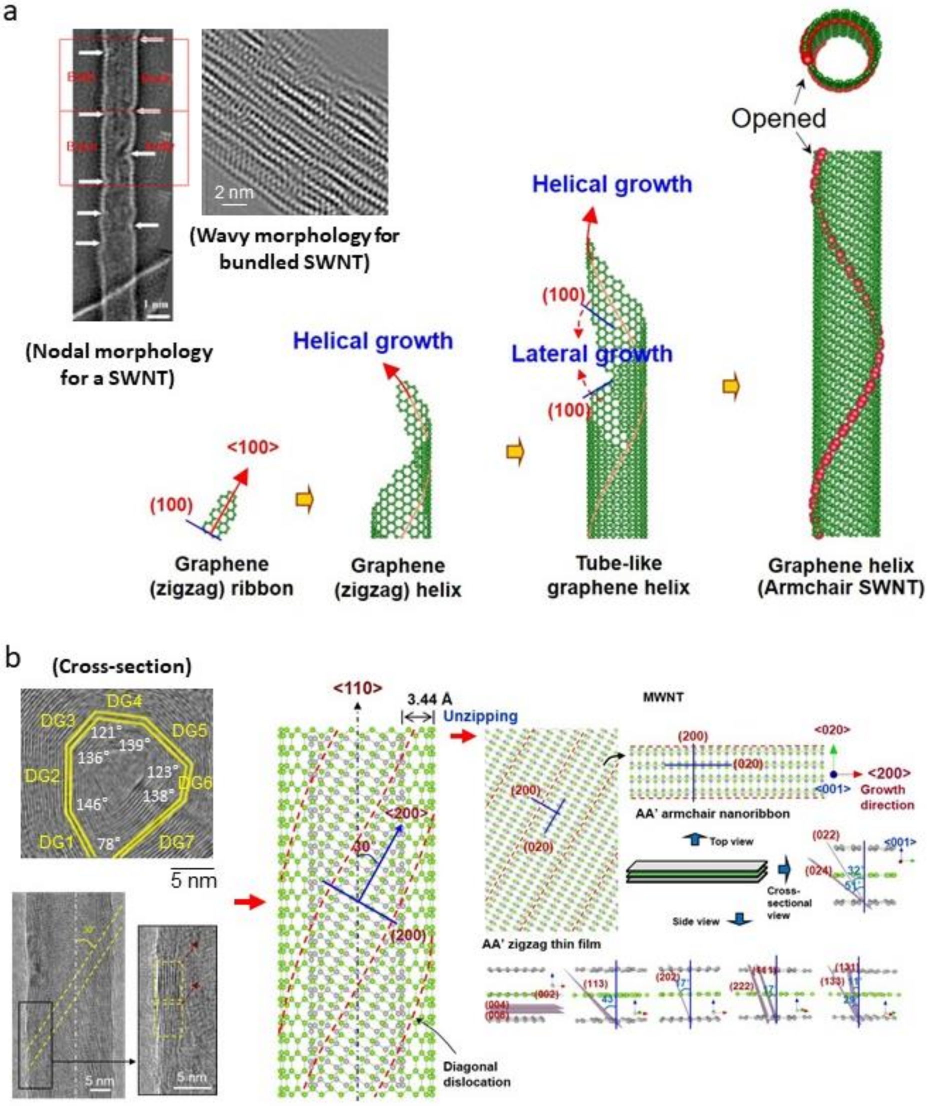

Figure S1. Structure models for SWNTs and MWNTs. (*a*) Structure model for SWNTs where an SWNT is formed by lateral growth following helical growth of a zigzag graphene ribbon (Lee *et al*., 2013). Nodal and wavy morphologies (HRTEM images) provide evidence for helical SWNTs (Lee *et al*., 2014). (*b*) Structure model of MWNTs. This model is based on HRTEM images for a CVD MWNT (HRTEM images), revealing plural stacks of graphite nanoribbons comprising the wall (Lee *et al*., 2013). The tubular structure can be simplified as a textured AA' thin film based on an AA' armchair nanoribbon. The cross-sectional HRTEM image, revealing a polygonal crystal (helix) with seven facets, also provides morphological evidence for helical MWNTs. We modelled the helix in this work. Helical growth (HG) comprising seven diagonal growths (DGs) results in a helix with a heptagon (seven-cornered), with internal angles ranging from 78° to 146° (regular angle of heptagon: 129°). The number of DGs in an HG may differ from sample to sample.

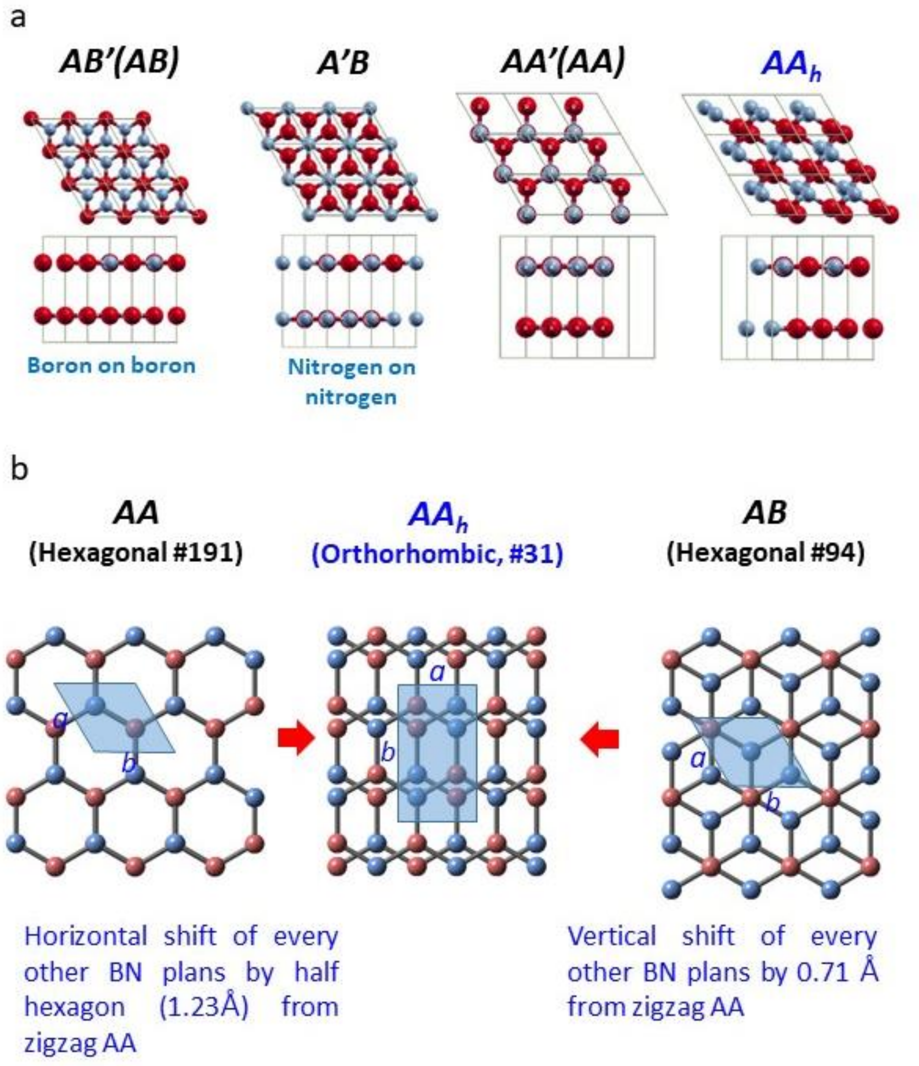

Figure S2. Stacking structures of BN and definition of AA_h BN. (*a*) Four typical stacking structures of BN. There is another BN stacking mode, *A′A*, which is unstable compared with *AA′* BN. (*b*) Crystallographic definition of AA_h BN. *AA*, *AB* and AA_h belong to #191 (*p6/mmm*, hexagonal), #194 (*p63/mmc*, hexagonal) and #31 ($Pm2_1$, orthorhombic), respectively. AA_h BN is defined as a horizontal shift of every other BN plane by a half hexagon (1.23 Å) relative to the *AA* zigzag structure or a vertical shift by a half interatomic distance (0.71 Å) relative to the *AB* zigzag structure.

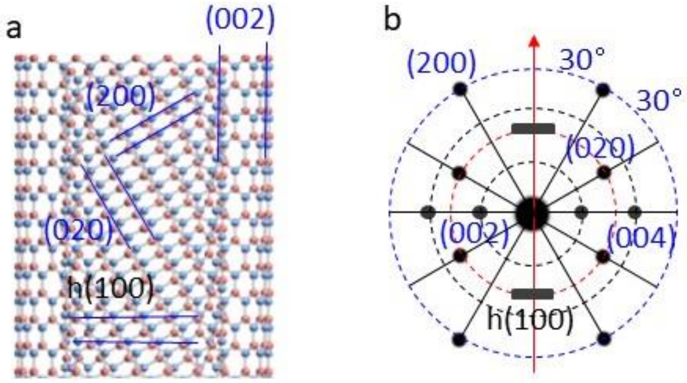

Figure S3. Structural model for a BNNT and its expected ED pattern. (*a*) AA_h BN-based structural model for BNNTs, where (002), (200), (020) and *h*(100) are represented. (*b*) ED pattern expected from the structural model in (*a*). *h*(100) appears due to local sliding of the layers of the tube walls, destroying the orthorhombic crystal but revealing (100) signals of the hexagonal crystal.

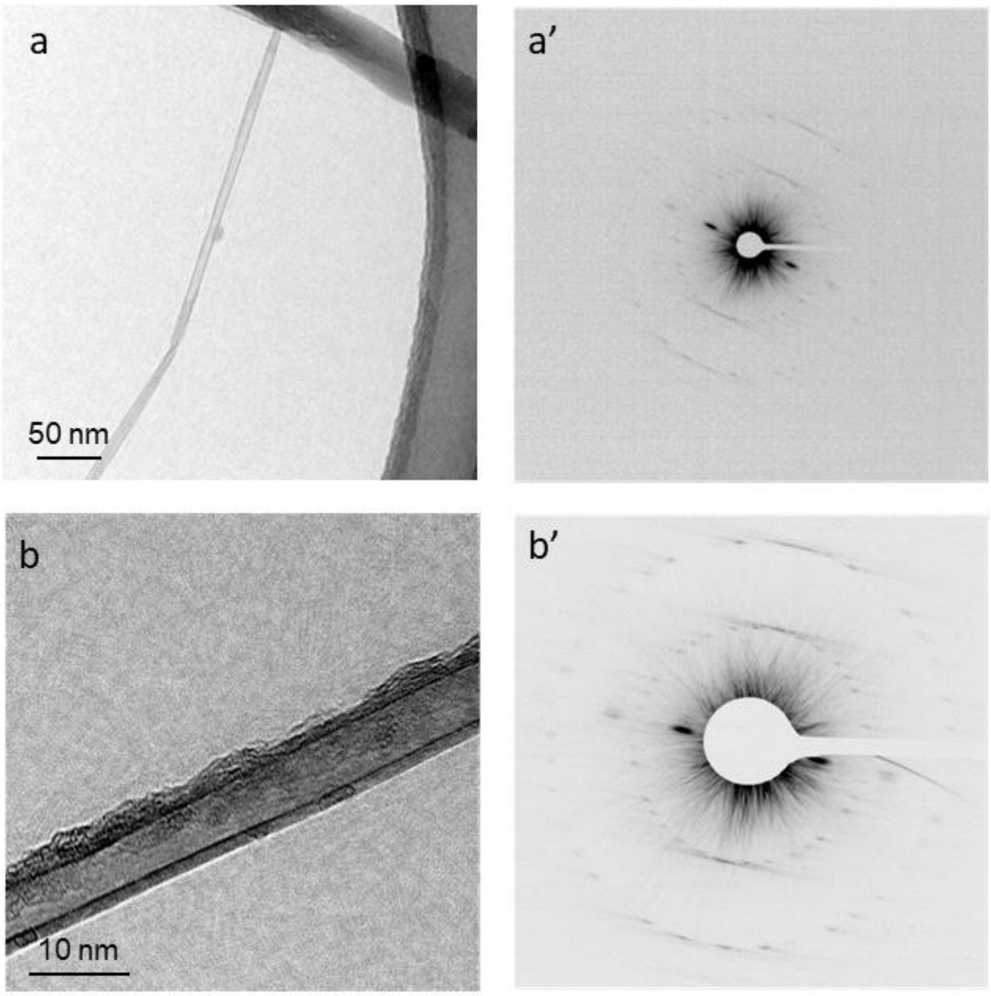

Figure S4. HRTEM and SAED patterns of BNNTs. (*a*, *a′*) A TEM image of a BNNT and its SAED pattern. (*b*, *b′*) HRTEM image of a BNNT and its SAED pattern. Both patterns reveal single-crystal features (spots) in a zigzag AA_h BN structure. The twin spots can be explained by the fact that the samples are twined (or double helix) and/or are lying tilted by ~3° horizontally on the TEM grid. The patterns are very similar to those reported for BNNTs.

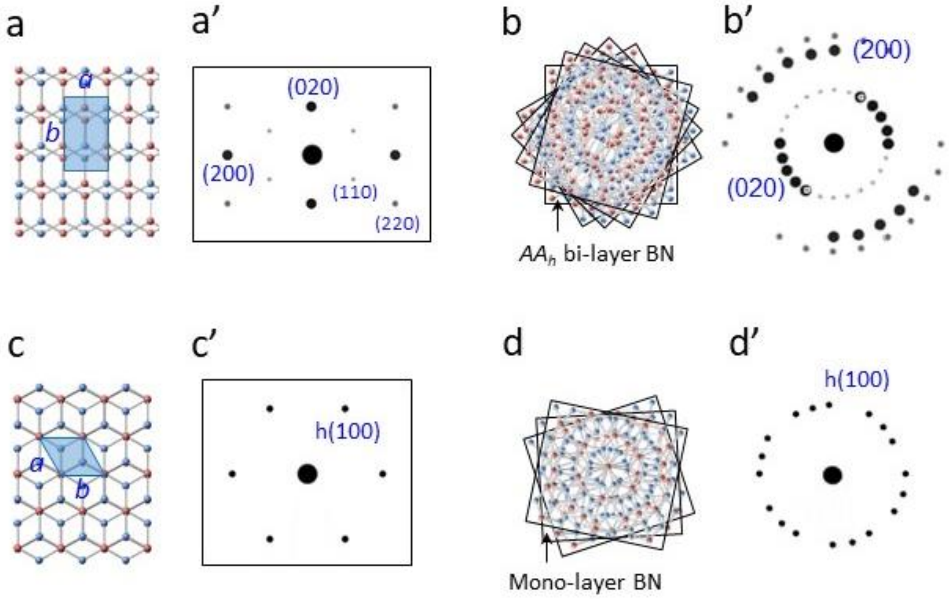

Figure S5. ED simulations. (*a*, *a′*) For the unit of AA_h BN (orthorhombic, #31 $Pm2_1$). (*b*, *b′*) For five AA_h bi-layer BN structures, where each is rotated by 15°. (*c*, *c′*) For the unit of *AB* BN (hexagonal, #94, $p6_3/mmc$). (*d*, *d′*) For three mono-layer BN structures, where each is rotated by 15°.

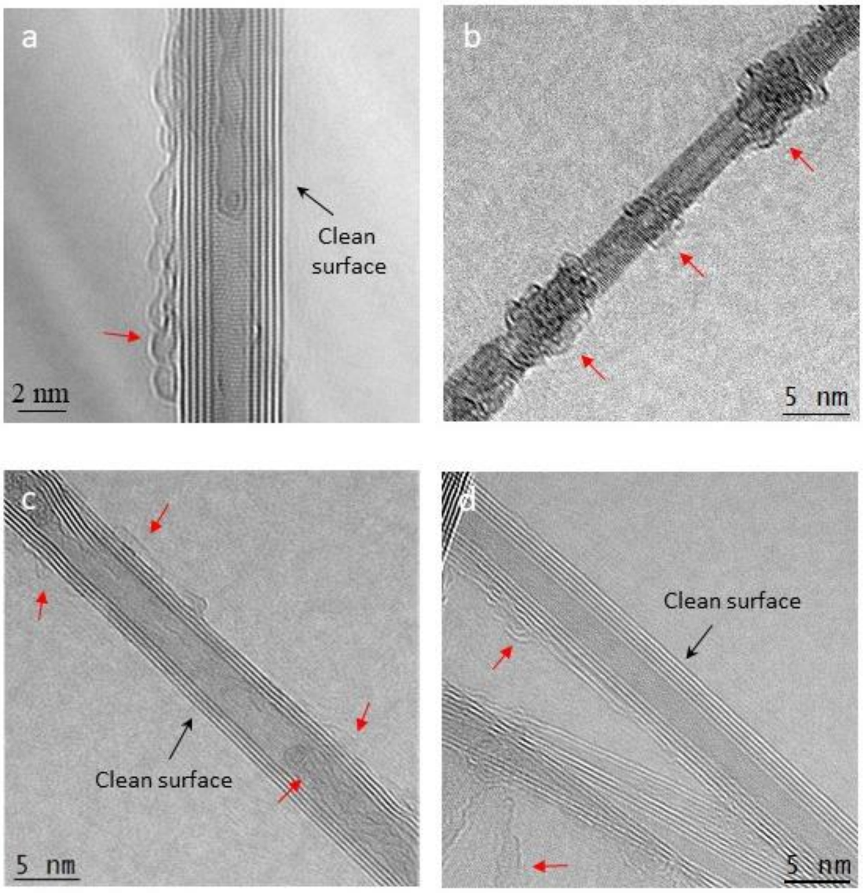

Figure S6. HRTEM images of BNNTs. The red arrows indicate the dominant mono-layer BN. The clean surface of BNNTs in (*a*, *c*, *d*) indicates that some samples (coherently scrolled) can be seen as concentric tubes.
